# Impact of COVID-19 first wave on the mental health of healthcare workers in a Front-Line Spanish Tertiary Hospital: lessons learned

**DOI:** 10.1038/s41598-024-58884-0

**Published:** 2024-04-08

**Authors:** Juan D. Molina, Franco Amigo, Gemma Vilagut, Philippe Mortier, Carmen Muñoz-Ruiperez, Irene Rodrigo Holgado, Alba Juanes González, Carolina Elisa Combarro Ripoll, Jordi Alonso, Gabriel Rubio

**Affiliations:** 1 Centro de Investigación Biomédica en Red (CIBER) Salud Mental, Madrid, Spain; 2 Villaverde Mental Health Center, Clinical Management Area of Psychiatry and Mental Health, Psychiatric Service, Hospital Universitario, 12 de Octubre, Av. de Córdoba S.N, Madrid, Spain; 3 Research Institute Hospital 12 de Octubre (I + 12), Madrid, Spain; 4 Facultad de Medicina, Universidad Francisco de Vitoria, Madrid, Spain; 5 Health Services Research Unit, Hospital del Mar Research Institute, Barcelona, Spain; 6 CIBER Epidemiología y Salud Pública (CIBERESP), Madrid, Spain; 7 Occupational Medicine and Occupational Risk Prevention Service, Hospital Universitario, 12 de Octubre, Madrid, Spain; 8 Clinic Psychologist, COVID-19 Assistance Project, 12 de Octubre University Hospital, Madrid, Spain; 9 Psychiatrist, COVID-19 Assistance Project, Consultation-Liaison Psychiatry Unit, 12 de Octubre University Hospital, Madrid, Spain; 10 Department of Experimental and Health Sciences, Pompeu Fabra University, Barcelona, Spain; 11 Department of Psychiatry, Faculty of Medicine, Complutense University of Madrid, Madrid, Spain; 12 Addictive Disorders Network, Redes Temáticas de Investigación Cooperativa (RETICS) (Thematic Networks of Cooperative Research in Health), Carlos III Health Institute, Ministerio de Ciencia e Innovación (MICINN) and Federación Española de Enfermedades Raras (FEDER), Madrid, Spain

**Keywords:** COVID-19, Mental health, Healthcare professionals, Psychiatric history, Microbiology, Psychology, Diseases

## Abstract

Healthcare workers (HCWs) were at high risk of experiencing psychological distress during COVID-19 pandemic. The objective of this study was to evaluate the impact on HCWs’ mental health in a Spanish hospital. Cross-sectional study of HCW, active between May and June 2020. A web-based survey assessed probable current mental disorders (major depressive disorder [PHQ-8 ≥ 10], generalized anxiety disorder [GAD-7 ≥ 10], panic attacks, post-traumatic stress disorder [PTSD; PLC-5 ≥ 7], or substance use disorder [CAGE-AID ≥ 2]). The Sheehan Disability Scale (SDS) was used to assess severe impairment and items taken from the modified self‐report version of the Columbia Suicide Severity Rating Scale (C‐SSRS) assessed suicidal thoughts and behaviors. A total of 870 HCWs completed the survey. Most frequent probable mental disorders were major depressive disorder (33.6%), generalized anxiety disorder (25.5%), panic attacks (26.9%), PTSD (27.2%), and substance use disorder (5.0%). Being female, having aged 18–29 years, being an auxiliary nurse, direct exposure to COVID-19-infected patients, and pre-pandemic lifetime mental disorders were positively associated with mental issues. Hospital HCWs presented a high prevalence of symptoms of mental disorders, especially depression, PTSD, panic attacks, and anxiety. Younger individuals and those with lifetime mental disorders have been more vulnerable to experiencing them.

## Introduction

In 2020, the COVID-19 outbreak was declared a pandemic by the World Health Organization (WHO), with 618 million and 13.8 million confirmed cases and 6.8 million and 119,618 deaths worldwide and in Spain, respectively as of March 2023^1,2^. Epidemiological studies have shown that past infectious diseases resulted in long-term and persistent psychological consequences among those affected^3–5^. Similarly, the COVID-19 pandemic has threatened global mental health, both indirectly via disruptive societal changes and directly via neuropsychiatric sequelae after SARS-CoV-2 infection^6^. Indeed, a previous Spanish study analyzing the clinical picture during the first wave by a factor analysis, showed that anosmia/ageusia, cognitive complaints, worry/nervousness, slowing down, and sadness have been the most frequently reported neuropsychological symptoms among hospitalized COVID-19 patients^7^. Therefore, in addition to its effects on the economy and public health, the COVID-19 pandemic has had a profound impact on the physical health and psychological well-being of healthcare workers (HCWs). HCWs have played a crucial role and are considered a high-risk population for suffering from psychological and mental disorders due to the COVID-19 pandemic^8^. The absence of data on transmission dynamics and evidence-based recommendations regarding the necessary protective measures made the situation particularly stressful for individuals employed in the healthcare sector. Moreover, HCWs were required to wear personal protective equipment, which can reduce their mobility and slow down their operations, can cause respiratory discomfort and difficulty, further exacerbating the psychological symptoms experienced by HCWs^9,10^. Several studies have documented that a significant proportion of HCWs experienced persistent psychological issues including anxiety, depression, and insomnia^9,11–13^. Spain ranked first in the world in healthcare infections during the first wave of the pandemic^14^. A nationwide, cross-sectional, web-based survey determined the mental health impact of the first wave of the COVID-19 pandemic (May–September 2020) on 9138 Spanish HCWs from 18 healthcare centers (MINDCOVID study)^15^. Authors revealed that approximately one in two HCWs experience an ongoing mental disorder, and 14.5% suffer a disabling one^15^. Another study by the MINDCOVID group, with data from 5450 HCWs of 10 Spanish hospitals between May and July 2020, reported a prevalence of 30-day suicidal thoughts and behaviors (STB) of 8.4%^16^.

The objective of the present study was to evaluate the impact of the COVID-19 pandemic on the mental health of HCWs during the first wave in one of the largest front-line tertiary hospitals from Spain and to explore potential factors associated with these probable mental disorders. These data would complement those previously reported in hospitalized COVID-19 patients, thus, providing a more detailed picture of the situation during the first wave of the COVID-19 pandemic in this Spanish hospital.

## Methods

### Study design

This is an observational analysis of data from HCWs at the University Hospital 12 de Octubre (Madrid, Spain). Information from HCWs represents a sub-analysis from the MINDCOVID study^15^. Institutional representatives from the hospital invited (by mail) all healthcare employees to participate in the study. All HCWs from the hospital with an institutional e-mail account were invited to participate, without any additional restriction. All participants accepted the online informed consent before being able to access the interview.

### Evaluation of probable mental health disorders

HCWs completed a web-based survey between May and June 2020 that included, among other items, standard screening instruments for measuring probable current mental disorders. Major depressive disorder (MDD) was assessed by the Spanish version of the Patient Health Questionnaire (PHQ-8), with the cut-off point ≥ 10 of the sum score^17,18^. Panic attacks were evaluated by assessing the number of attacks in the 30 days prior to the interview^19,20^. For evaluating post-traumatic stress disorder (PTSD), the 4-item version of the PTSD checklist for DSM-5 (PCL-5) was applied^20,21^. Generalized anxiety disorder (GAD) was evaluated by using the Spanish adaptation of the 7-item GAD scale (GAD-7), with a cut-off point of ≥ 10^19,22–24^. Substance Use Disorder (SUD) was evaluated by using the Spanish version of the CAGE questionnaire adapted to include drugs (CAGE-AID) with a cut-off point of ≥ 2^25,26^. Any of the above probable mental disorders was considered “disabling” if the participant reported severe role impairment (score ≥ 7) during the past 12 months according to an adapted version of the Sheehan Disability Scale^27–29^. Lifetime mental disorders, prior to the COVID-19 pandemic, were assessed by using a checklist based on the Composite International Diagnostic Interview (CIDI) that screens for self-reported lifetime depressive disorder, bipolar disorder, anxiety disorders, panic attacks, alcohol and drug use disorders, and “other” mental disorders. More information from the survey instrument is provided elsewhere^15^. A modified self‐report version of selected items from the Columbia Suicide Severity Rating Scale (C‐SSRS) was applied to assess suicidial thoughts and behaviors in the past 30 days^30^, including passive suicidal ideation (“wish you were dead or would go to sleep and never wake up”), active suicidal ideation (“have thoughts of killing yourself”), suicide plans (“think about how you might kill yourself [e.g., taking pills, shooting yourself] or work out a plan of how to kill yourself”), and suicide attempt (“make a suicide attempt [i.e., purposefully hurt yourself with at least some intent to die”).

### Statistical analysis

Demographic, clinical, and survey characteristics of HCWs are expressed with the mean and standard deviation (SD), or with absolute and relative frequencies, when appropriate. Post-stratification weights were applied with raking procedure to restore distributions the elegible personnel within the hospital according to age, gender and professional category. Statistical significance was assessed with pooled Chi-square test from multiple imputations after adjustment for multiple comparisons with Benjamin-Hochberg (false discovery rate 0.05). Missing item-level data among respondents were imputed using multiple imputation by chained equations with 12 imputed datasets and 10 iterations per imputation^31^.

### Ethical approval and informed consent

The study complies with the Declaration of Helsinki and the Code of Ethics and was approved by the IRB Parc de Salut Mar (2020/9203/I) and by the corresponding IRBs of all the participating centres. Registered at ClinicalTrials.gov (https://clinicaltrials.gov/ct2/show/NCT04556565). The database was anonymized to preserve sensitive data from patients. Participants signed the written informed consent.

## Results

### Participants

Of the 7797 eligible professionals, 870 HCWs participated and completed the survey (Sociodemographics are shown in Table 1). The response rate, calculated as the number of participants that completed it divided by the estimated eligible workers, was 11.8%. Survey participation (workers that agreed to participate divided by those who responded to the informed consent) and completion rates (participants that completed the survey divided by those who agreed to participate) were 88.5% and 78.9%, respectively. HCWs of the sample were predominantly females (78.1%), with a mean age of 43.7 (SD: 11.4), and working mainly as nurses (28.1%), physicians (18.3%), and auxiliary nurses (18.3%). Half of them (52.3%) were frontline workers during the COVID-19 pandemic. Most of them were not infected (77.1%) at the time of the study. The most frequent previous lifetime mental disorder was anxiety (41.7% of them), followed by mood disorder (12.8%).

**Table 1:** Sociodemographic characteristics and COVID-related factors of the sample of healthcare workers (HCWs) from the University Hospital 12 Octubre (Madrid, Spain).

	Healthcare workers N (%)
Total	870 (100)
Gender, n (%)	
Male	170 (21.9)
Female	700 (78.1)
Age, mean years (SD)	43.7 (11.4)
Groups, n (%)	
18–29 years	133 (13.6)
30–49 years	432 (47.2)
> 50 years	305 (39.2)
Position, n (%)	
Physician	217 (18.3)
Nurse	260 (28.1)
Auxiliary nurse	105 (18.3)
Others involved in patient’s care	118 (12.6)
Others not involved in patient’s care	170 (22.6)
Frontline work during COVID-19, n (%)	
Yes	450 (52.3)
No	420 (47.7)
COVID-19 infection history, n (%)	
Not Infected	666 (77.1)
Positive (test or symptoms)	195 (21.6)
Positive and hospitalized	9 (1.2)
Lifetime disorders, n (%)	
Anxiety disorder	351 (41.7)
Mood disorder	106 (12.8)
Substance use disorder	7 (0.8)
Any mental disorder	406 (48.1)

SD: Standard deviation; n: number of healthcare workers; %: porcentage.

### Probable mental disorders

**Figure 1:**
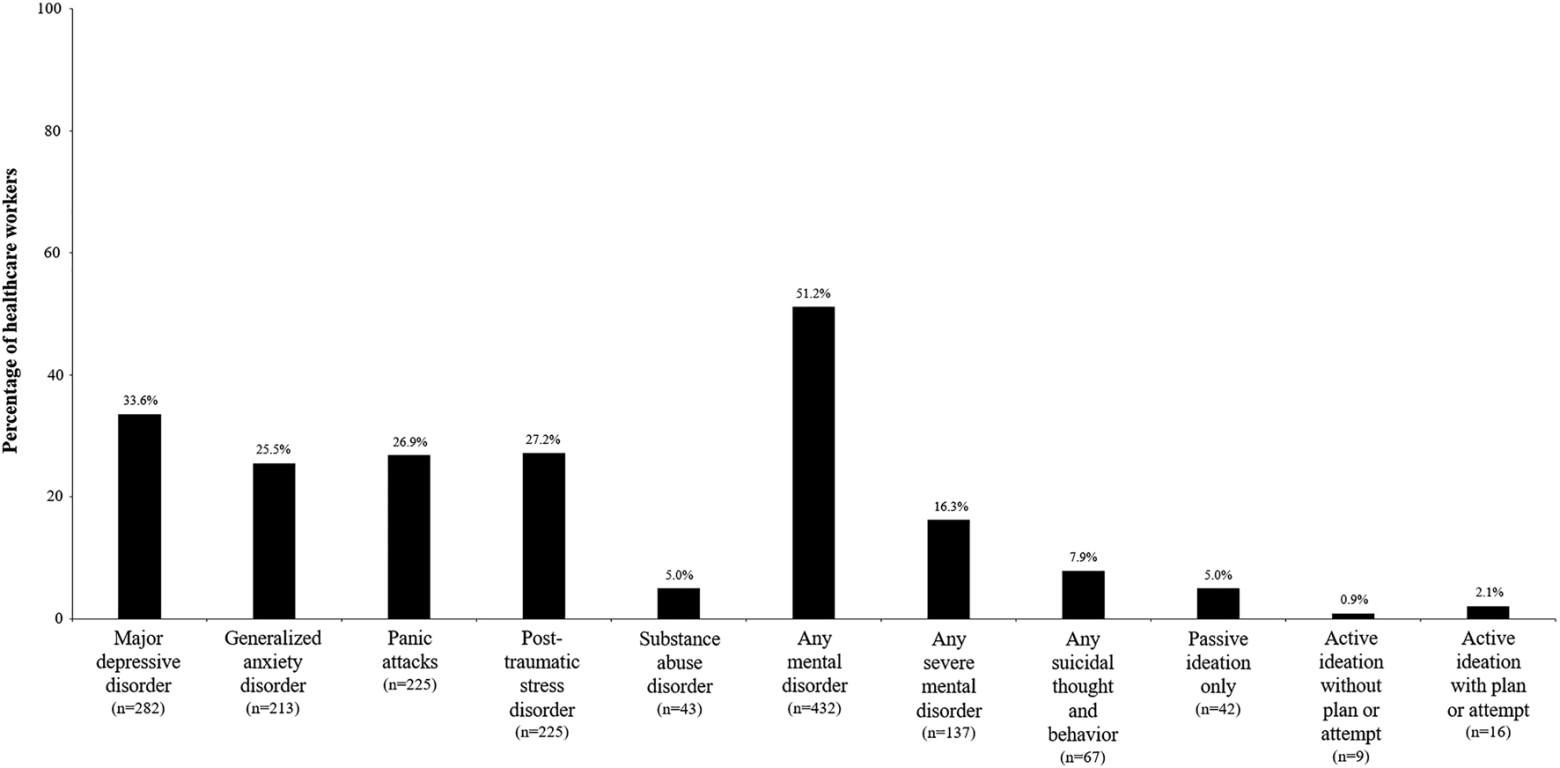
Prevalence of probable mental disorders and STBs among healthcare workers from the University Hospital 12 de Octubre (Madrid, Spain).

The most frequent probable mental disorders identified in HCWs were MDD (33.6% of them), GAD (25.5%), panic attacks (26.9%), PTSD (27.2%), and SUD (5.0%) (Fig. 1). In total, 51.2% of the HCWs had a probable current mental disorder, and 16.3% presented a disabling mental disorder. The prevalence of any suicidal thought and behavior (STB) was 7.9% (Fig. 1).

Table 2 shows the prevalence of all mental disorders and STBs according to sociodemographic variables and by proximal and distal risk factors. Significant differences (higher prevalence) of mental disorders were found regarding gender (females, for current MDD, GAD, panic attacks, PTSD, and any mental and any disabling mental disorder); age (18–29 years, for current MDD, GAD, panic attacks, PTSD, any mental disorder, and any STB); job position (auxiliary nurse, for current MDD, GAD, panic attacks, PTSD, and any previous mental and disabling mental disorder); direct exposure to infected patients (for current MDD, GAD, panic attacks, PTSD, and any mental disorder); and lifetime disorders (mood, anxiety, other mental disorder) (Table 2).

**Table 2:** Probable mental disorders and STBs according to sociodemographic and COVID-19 characteristics of HCWs (N = 870) from the University Hospital 12 de Octubre (Madrid, Spain).

	Current MDD (n = 282)	Current GAD (n = 213)	Current panic attacks (n = 225)	Current PTSD (n = 225)	Current substance use disorder (n = 43)	Any current mental disorder (n = 432)	Any current severe mental disorder (n = 137)	Any STB (n = 67)	Passive ideation only (n = 42)	Active ideation without plan of attempt (n = 9)	Active ideation with plan or attempt (n = 16)
	%^a^ (SE)	%^a^ (SE)	%^a^ (SE)	%^a^ (SE)	%^a^ (SE)	%^a^ (SE)	%^a^ (SE)	%^a^ (SE)	%^a^ (SE)	%^a^ (SE)	%^a^ (SE)
Gender											
Male	22.6 (3.5)*	15.9 (3.1)*	14.1 (3.0)*	14.6 (3.0)*	8.6 (2.4)	38.0 (4.1)*	6.9 (2.4)*	8.9 (2.4)	5.6 (1.9)	0.9 (0.8)	2.5 (1.3)
Female	36.6 (2.2)	28.2 (2.0)	30.4 (2.1)	30.7 (2.1)	4.0 (0.9)	54.9 (2.2)	18.9 (1.9)	7.7 (1.2)	4.8 (1.0)	0.9 (0.4)	2.0 (0.6)
Age											
18–29 years	50.4 (5.3)*	38.4 (5.2)*	39.0 (5.2)*	41.7 (5.3)*	6.7 (2.7)	68.1 (5.0)*	21.3 (5.0)	17.5 (4.0)*	9.1 (3.1)	3.1 (1.8)	5.3 (2.4)
30–49 year	31.0 (2.7)	25.7 (2.5)	29.1 (2.6)	26.6 (2.5)	5.9 (1.4)	50.0 (2.9)	18.7 (2.4)	4.6 (1.2)	2.7 (0.9)	0.7 (0.5)	1.2 (0.6)
50 years or more	30.8 (2.9)	20.9 (2.6)	19.9 (2.5)	22.7 (2.6)	3.3 (1.2)	46.7 (3.1)	11.7 (2.3)	8.6 (1.8)	6.2 (1.5)	0.4 (0.4)	2.0 (0.9)
Position											
Physician	21.8 (3.9)*	14.8 (3.3)*	10.0 (2.8)*	10.5 (2.9)*	7.7 (2.5)	31.8 (4.3)*	5.8 (2.4)*	6.8 (2.3)	2.6 (1.5)	2.2 (1.4)	1.9 (1.3)
Nurse	38.5 (3.6)	27.9 (3.3)	27.8 (3.4)	30.6 (3.4)	3.8 (1.4)	58.9 (3.7)	21.5 (3.3)	6.3 (1.8)	5.2 (1.7)	0.3 (0.4)	0.8 (0.7)
Auxiliary nurse	51.4 (4.6)	40.9 (4.5)	42.8 (4.6)	43.6 (4.6)	6.4 (2.3)	67.8 (4.3)	26.6 (4.6)	9.4 (2.7)	4.0 (1.8)	0.8 (0.8)	4.5 (1.9)
Other profession involved in patient care	25.5 (4.8)	24.2 (4.7)	33.7 (5.3)	28.0 (5.0)	2.7 (1.8)	44.9 (5.5)	16.0 (4.4)	9.2 (3.2)	5.8 (2.6)	0.7 (0.9)	2.7 (1.8)
Other professions not involved in patient care	27.0 (3.7)	19.6 (3.3)	22.5 (3.5)	22.5 (3.5)	4.4 (1.8)	47.4 (4.2)	10.2 (2.8)	9.0 (2.4)	6.9 (2.2)	0.7 (0.7)	1.4 (1.0)
Work during COVID-19											
Frontline	41.6 (2.7)*	32.0 (2.6)*	32.6 (2.6)*	33.8 (2.6)*	6.3 (1.3)	59.0 (2.7)*	18.9 (2.3)	9.3 (1.6)	6.2 (1.3)	0.8 (0.5)	2.4 (0.9)
Not-frontline	24.8 (2.5)	18.5 (2.2)	20.6 (2.3)	19.9 (2.3)	3.6 (1.1)	42.6 (2.8)	13.4 (2.2)	6.4 (1.4)	3.7 (1.1)	1.1 (0.6)	1.7 (0.8)
Frequency of direct exposure to COVID-19											
All of the time	44.4 (3.5)*	38.0 (3.4)*	36.1 (3.4)*	40.7 (3.5)*	6.5 (1.8)	64.4 (3.4)*	22.0 (3.2)	11.3 (2.2)	7.7 (1.9)	0.7 (0.6)	2.9 (1.2)
Most of the time	37.1 (4.2)	22.6 (3.7)	27.1 (3.9)	23.1 (3.7)	5.8 (2.1)	50.8 (4.4)	14.2 (3.3)	6.2 (2.2)	3.7 (1.7)	0.9 (0.8)	1.6 (1.1)
Some of the time	29.6 (3.6)	21.7 (3.2)	20.7 (3.2)	23.9 (3.3)	3.7 (1.5)	48.0 (3.9)	14.5 (3.0)	8.1 (2.2)	4.4 (1.7)	1.4 (0.9)	2.3 (1.2)
A little of the time	17.5 (4.4)	14.3 (4.1)	24.2 (4.9)	15.3 (4.2)	5.1 (2.5)	37.4 (5.5)	11.9 (4.2)	2.7 (1.9)	1.0 (1.3)	0.6 (0.9)	1.1 (1.2)
None of the time	21.2 (5.3)	15.0 (4.7)	15.8 (4.7)	15.0 (4.7)	1.3 (1.5)	34.6 (6.1)	12.4 (4.5)	6.8 (3.4)	5.0 (2.9)	0.7 (1.1)	1.0 (1.3)
Lifetime mood disorder											
No	31.0 (2.0)*	23.7 (1.8)*	25.7 (1.9)	25.8 (1.9)	4.4 (0.9)	48.5 (2.1)*	14.5 (1.7)*	6.3 (1.0)*	4.1 (0.8)	0.7 (0.3)	1.5 (0.5)
Yes	50.9 (5.5)	38.0 (5.4)	34.6 (5.3)	36.5 (5.3)	9.0 (3.2)	69.6 (5.1)	28.3 (5.5)	19.4 (4.4)	10.7 (3.4)	2.7 (1.8)	6.0 (2.7)
Lifetime anxiety disorder											
No	23.8 (2.2)*	16.6 (1.9)*	17.5 (2.0)*	19.7 (2.1)*	3.4 (1.0)	39.1 (2.5)*	10.3 (1.8)*	5.1 (1.1)*	3.2 (0.9)	0.5 (0.4)	1.3 (0.6)
Yes	47.3 (3.1)	38.1 (3.0)	39.9 (3.0)	37.6 (3.0)	7.2 (1.6)	68.1 (2.9)	24.7 (3.0)	11.9 (2.0)	7.4 (1.6)	1.5 (0.8)	3.1 (1.1)
Lifetime SUD											
No	33.4 (1.9)	25.5 (1.7)	26.9 (1.8)	27.2 (1.8)	4.7 (0.9)*	51.0 (2.0)	16.3 (1.6)	7.9 (1.1)	5.0 (0.9)	0.9 (0.4)	2.0 (0.6)
Yes	57.7 (22.0)	24.2 (19.1)	26.4 (19.7)	23.9 (19.0)	45.5 (22.1)	69.7 (20.5)	11.8 (14.4)	11.5 (14.2)	0.0 (0.0)	0.0 (0.0)	11.5 (14.2)
Other lifetime mental disorders											
No	32.6 (1.9)*	25.0 (1.7)	26.3 (1.8)	26.9 (1.8)	4.8 (0.9)	50.3 (2.0)*	15.5 (1.6)*	7.6 (1.1)	4.7 (0.9)	0.9 (0.4)	2.0 (0.6)
Yes	64.6 (10.9)	42.1 (11.2)	45.1 (11.4)	34.9 (10.9)	10.4 (7.3)	81.3 (9.0)	41.9 (13.2)	18.6 (8.9)	13.8 (7.9)	0.0 (0.0)	4.8 (4.9)
Any lifetime mental disorder											
No	21.2 (2.2)	15.0 (2.0)	16.1 (2.1)	18.1 (2.1)	2.9 (0.9)	35.9 (2.7)	8.3 (1.7)	4.4 (1.1)	3.4 (1.0)	0.6 (0.4)	0.5 (0.4)
Yes	46.9 (2.8)*	36.9 (2.7)*	38.4 (2.8)*	36.9 (2.7)*	7.3 (1.5)	67.7 (2.7)*	24.9 (2.7)*	11.7 (1.8)*	6.7 (1.4)	1.3 (0.7)	3.7 (1.1)

GAD: Generalized anxiety disorder; MDD: major depressive disorder; PTSD: post-traumatic stress disorder; SE: standard error; STB: suicidal thoughts and behaviours.

*Pooled Chi-square test from multiple imputations statistically significant after adjustment for multiple comparisons with Benjamini-Hochberg (false discovery rate 0.05).

^a^Weighted percentage (using post-stratification weights obtained with raking procedure).

## Discussion

The emergence of the COVID-19 pandemic has led to a worldwide public health emergency, causing significant psychological challenges in the global healthcare system, particularly during the initial stages of the outbreak^32^. The results obtained from the present study show a high prevalence of current probable mental disorders and suicidal ideation in a large sample of HCWs from Hospital 12 de Octubre (Spain) during the first wave of the COVID-19 pandemic. Symptoms of MDD were the most frequently reported, followed by GAD, panic attacks, and PTSD. Furthermore, we observed that HCWs with lifetime mental disorders had a notably higher occurrence of adverse mental health. Other specific variables such as gender, age, job position, and direct exposure to infected patients were found to pose a greater risk in the onset of mental disorders.

During the COVID-19 pandemic, HCWs have been confronted with an unprecedented situation that has taken a toll on their mental and physical health^33^. Their essential duties required them to make difficult decisions under extreme pressure, thereby placing them at a higher risk of developing mental health disorders^34,35^. Previous studies evidencing the impact of the COVID-19 crisis on the mental health of HCWs are in agreement with the findings presented herein^15,16,36–39^. A survey carried out in May 2020 by the British Medical Association showed that 45% of UK physicians were experiencing anxiety, depression, stress, or other mental issues due to the COVID-19 pandemic^36^. In Spain, another survey performed during the first COVID-19 wave (between May 2020 and September 2020), reported that 43.7% of the 2929 primary care professionals (95% confidence interval [CI] = 41.9–45.4) screened positive for a probable mental disorder^37^. In general, published studies evaluating the psychological toll on HCWs report symptoms of anxiety, depression, insomnia, or distress^38^. The prevalence of depressive symptoms and anxiety ranges from 8.9 to 50.4% and between 14.5 and 44.6%, respectively. A systematic review and meta-analysis, involving data from 70 studies and 101,017 HCWs, have revealed a pooled prevalence of 30.0% for anxiety, 31.1% for depression and depressive symptoms, 56.5% for acute stress, 20.2% for post-traumatic stress, 44.0% for sleep disorders^39^. Another systematic review and meta-analysis, including 12 studies related to the psychological impact of the COVID-19 outbreak on HCWs in Asian countries, reported an overall prevalence rate of anxiety, depression, and stress of 34.8%, 32.4% and 54.1%, respectively^40^.

In Spain, MINDCOVID studies were multicenter observational trials that aimed to evaluate the impact of COVID-19 on the mental well-being of HCWs during the first wave^15,16^. In line with our findings, almost half of the surveyed workers (45.7%) presented any current mental disorder and 14.5% tested positive for a disabling mental disorder^15^. The most frequent probable mental disorders reported were current MDD (28.1%), GAD (22.5%), panic attacks (24.0%), PTSD (22.2%), SUD (6.2%), and any STB (8.4%). Other prevalence rates reported were passive ideation (4.9%), active ideation without plan or attempt (0.8%), and active ideation with plan or attempt (2.7%)^15,16^. When comparing our present results (unicenter) with those from the multicenter, nationwide MINDCOVID study^15^, prevalence rates of any probable mental disorder, MDD and PTSD are higher (Supplementary Fig. 1). The observed differences may respond to the different casuistry of Spanish hospitals during the first wave. Indeed, the pandemic carried out potentially traumatic moral and ethical challenges (e.g., choosing whom to indicate a ventilator in a situation where there was not for everyone) that exposed HCWs to the risk of developing moral injury^41,42^. Although moral injury is not considered a mental disorder yet, it is thought to be associated with PTSD by symptomatology and etiology, since both could be two different responses to trauma^41,42^. Thus, the higher prevalence of this PTSD could be attributed to the exposure of HCWs to these moral stressors^41,42^.

Regarding potential factors associated with probable mental disorders, our study pointed out a higher vulnerability of young individuals (aged between 18 and 29 years), female gender, and those with lifetime mental disorders (*p* < 0.05). Sociodemographic factors, such as gender and age have been previously related to a higher risk^38^. Female sex has been associated with a increased risk of mental disorders in several studies analyzing the impact of the COVID-19 pandemic on the mental health of HCWs^43–47^. A recent meta-analysis conducted by Lee and colleagues including 401 studies, has reported higher odds of probable mental health disorders in women, in particular depression, anxiety, PTSD and insomnia^48^. Multiple explanations or mechanisms have been proposed to explain these differences, including potential response bias (e.g., males may experience greater difficulty in recognizing and expressing psychological distress) as well as various biological, social, and demographic factors^49,50^. Therefore, although age and gender appear to be risk factors, this should be considered with caution. Moreover, the existence of a previous mental disorder has been identified as a predictor of other mental issues, such as depression and anxiety, during COVID-19^51^. Also, some studies have indicated that the COVID-19 pandemic could have a negative impact on current mental disorders^52^. Given that all HCWs were exposed to a high risk of developing or aggravating psychiatric symptoms, those with prior or current mental disorders would have been more vulnerable during the COVID-19 pandemic.

The limited availability of personal protective equipment, the continuous exposure to infected patients, the rate of deaths, the absence of specific treatments, overwhelming workload are other factors contributing to the development of these mental issues^53^. Additionally, HCWs' rising anxiety about the spread of COVID-19 may be linked to the misinformation that circulated during the initial wave of the pandemic and the concern that they may be a possible risk of contagion to their partner and family^43,54^. Furthermore, herein it was observed that some job positions, specifically auxiliary nurses, have a higher risk of mental disorders (*p* < 0.05). Maunder et al.^55^ studied the trend of burnout and psychological distress among HCWs from the fall of 2020 to the summer of 2021 and also found that nurses mostly reported the highest rates of burnout. Similarly, Fattori et al.^56^ observed that nurses and health assistants had higher risks of scoring above cut-offs than physicians (OR = 4.72 and 6.76 respectively). Differences between public and private healthcare sectors has been also analyze previously. According to a recent study by Pabón-Carrasco, HCWs employed in publicly healthcare institutions reported a lower perceived risk of COVID-19 transmission compared to their counterparts in private institutions during the first wave^43^. However, anxiety levels were higher in public employed HCWs compared to those reported by those privately employed (more than 25% and ~ 20%, respectively). Both groups had high levels of anxiety, despite private sector was not considered first-line^43^.

Some limitations of our study should be considered. First, its cross-sectional design, without similar information collected before the pandemic, does not allow us to infer the causality of the impact of the COVID-19 pandemic on the mental health of HCWs, nor to estimate the true magnitude of change in the prevalence of probable mental disorders. Furthermore, it is worth mentioning that during the first wave of the pandemic, psychological support was offered on demand to those professionals who requested it voluntarily at the hospital. Additionally, group interventions were conducted to alleviate symptoms at the onset. It would have been interesting to assess the impact of these interventions as a protective factor; however, we lack this data, which constitutes an additional limitation and possible bias.

Second, the response rate was lower than expected. It is possible that those experiencing mental health issues were more willing to participate or stressed workers did not have time to respond. However, weighting data has attempted to counteract this limitation. Third, this study’s assessments are based on self-reports from HCWs and not clinically diagnosed mental disorders. It is for this reason that we describe them as probable mental disorders.

Importantly, our approach has been used in most epidemiological studies, allowing for comparisons of results^21,23,57^. A more detailed analysis of proximal factors would have been interesting for linking the probable mental disorders with pandemic-related stressors.

Despite the above-mentioned limitations, we are confident to conclude that, during the first wave of the COVID-19 pandemic, HCWs of this large Spanish university hospital have presented a high prevalence of probable mental disorders, especially depression, PTSD, panic attack and anxiety. Younger individuals and those with lifetime mental disorders have been more vulnerable to experiencing them.

Based on our results, it appears to be expected that there is a significant demand for mental healthcare services among healthcare professionals in this Hospital that needs to be addressed. Our results, like others, highlight the significance of closely monitoring the psychological well-being of HCWs and facilitating their access to psychological assistance.

Understanding this data can also be relevant when selecting profiles of professionals who should be especially protected in high-stress situations, in order to care for their emotional well-being, such as those workers with a history of mental health issues or other vulnerability factors.

Future studies are needed to determine the evolution of the psychological impact of the COVID-19 pandemic over time in HCWs to implement appropriate therapeutic interventions.

### Supplementary Information

Supplementary Information.

## Data Availability

The de‐identified participant data as well as the study protocol and statistical analysis plan used for this study are available upon reasonable request from the corresponding author as long as the main objective of the data sharing request is replicating the analysis and findings as reported in this paper.
