# Supplementary material for: Impact of COVID-19 first wave on the mental health of healthcare workers in a Front-Line Spanish Tertiary Hospital: lessons learned
Source: Sci Rep. 2024 Apr 8;14:8149. doi: 10.1038/s41598-024-58884-0 (PMC11001893; doi:10.1038/s41598-024-58884-0)
Supplement: Supplementary file 1 — Supplementary Information. [file 41598_2024_58884_MOESM1_ESM.pptx]

## Slide 1
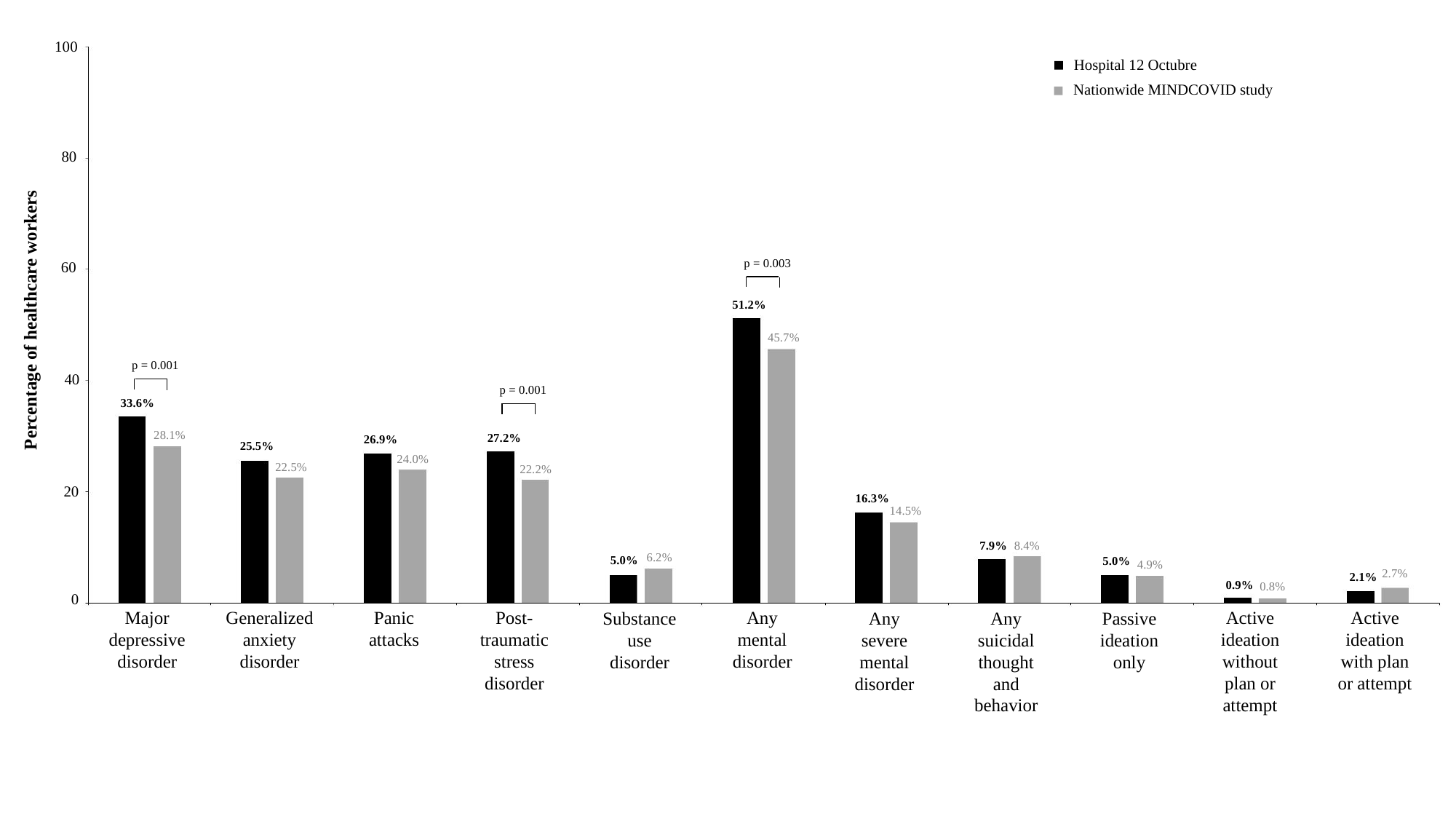

100
Hospital 12 Octubre
Nationwide MINDCOVID study
80
p = 0.003
60
51.2%
Percentage of healthcare workers
45.7%
p = 0.001
40
p = 0.001
33.6%
28.1%
27.2%
26.9%
25.5%
24.0%
22.5%
22.2%
20
16.3%
14.5%
7.9%
8.4%
6.2%
5.0%
5.0%
4.9%
2.7%
2.1%
0.9%
0.8%
0
Panic attacks
Post-traumatic stress disorder
Any mental
disorder
Major depressive disorder
Generalized anxiety disorder
Active ideation without plan or attempt
Active ideation with plan or attempt
Any suicidal
thought and behavior
Passive ideation only
Substance use disorder
Any severe mental
disorder

## Slide 2
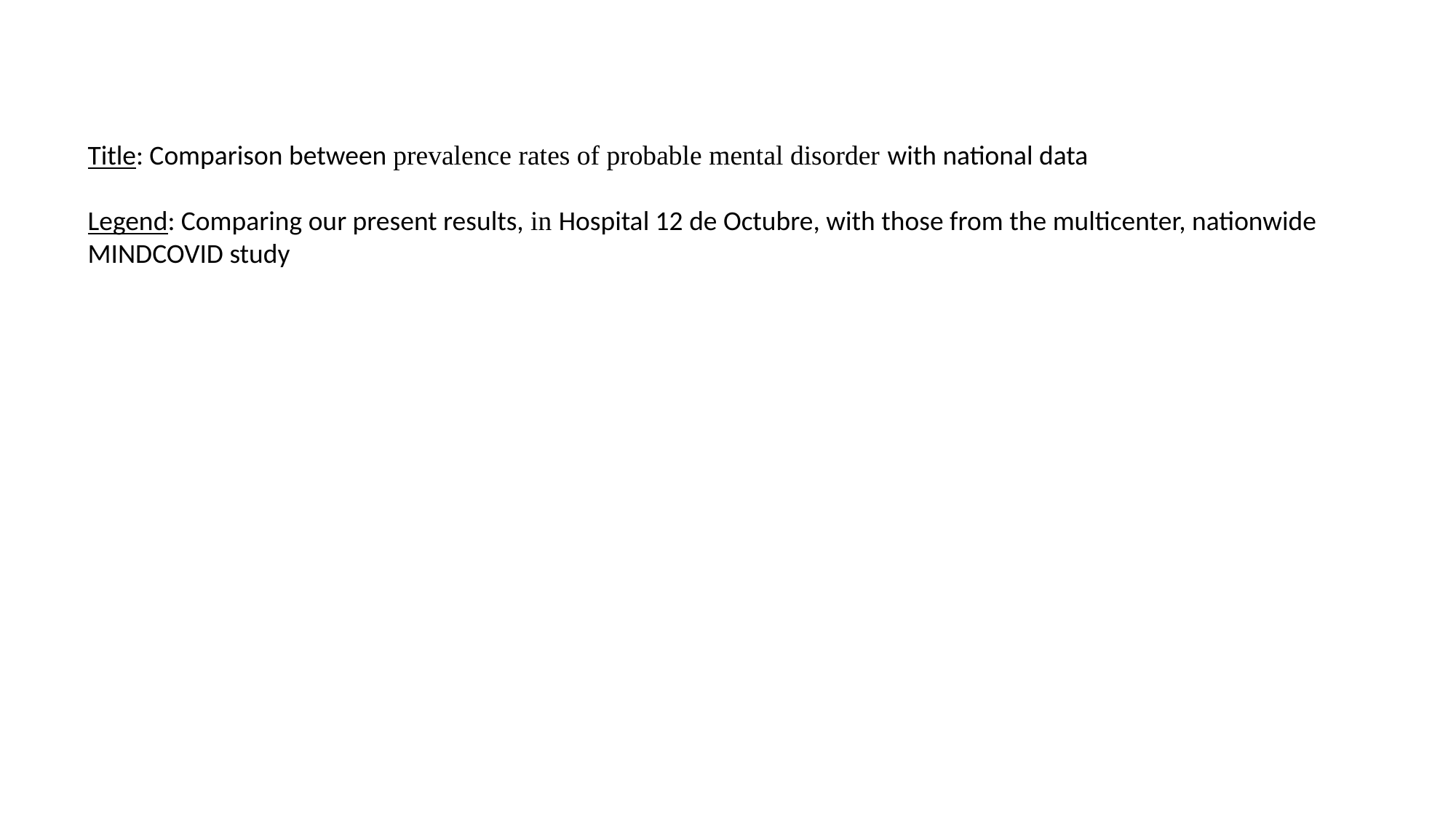

Title: Comparison between prevalence rates of probable mental disorder with national data
Legend: Comparing our present results, in Hospital 12 de Octubre, with those from the multicenter, nationwide MINDCOVID study
